# Microscopic Analysis of Bacterial Inoculum Effect Using Micropatterned Biochip

**DOI:** 10.3390/antibiotics10030300

**Published:** 2021-03-13

**Authors:** Jung Ho Hwang, Sang Young Lee, Jungil Choi

**Affiliations:** 1 Department of Electrical and Computer Engineering, Undergraduate School, Michigan State University, 426 Auditorium Road, East Lansing, MI 48824, USA; hwangj18@msu.edu; 2 Department of Mechanical Engineering, Undergraduate School, Kookmin University, 77 Jeongneung-ro, Seongbuk-gu, Seoul 02707, Korea; ehekfpfk@kookmin.ac.kr; 3 School of Mechanical Engineering, Kookmin University, 77 Jeongneung-ro, Seongbuk-gu, Seoul 02707, Korea

**Keywords:** inoculum effect, image-based AST, minimal inhibitory concentration, antibiotic susceptibility testing

## Abstract

Antimicrobial resistance has become a major problem in public health and clinical environments. Against this background, antibiotic susceptibility testing (AST) has become necessary to cure diseases in an appropriate and timely manner as it indicates the necessary concentration of antibiotics. Recently, microfluidic based rapid AST methods using microscopic analysis have been shown to reduce the time needed for the determination of the proper antibiotics. However, owing to the inoculum effect, the accurate measurement of the minimal inhibitory concentration (MIC) is difficult. We tested four standard bacteria: *Staphylococcus aureus, Pseudomonas aeruginosa, Escherichia coli, and Enterococcus faecalis*, against five different antibiotics: piperacillin, cefotaxime, amikacin, levofloxacin, and ampicillin. The results showed that overall, the microfluidic system has a similar inoculum effect compared to the conventional AST method. However, due to the different testing conditions and determination protocols of the growth of the microfluidic based rapid AST, a few results are not identical to the conventional methods using optical density. This result suggests that microfluidic based rapid AST methods require further research on the inoculum effect for practical use in hospitals and can then be used for effective antibiotic prescriptions.

## 1. Introduction

The emergence of antimicrobial resistance is a major issue globally. The pathogens exhibiting this resistance are called “superbugs,” which pose a huge threat not only in hospitals but also in the community [1,2]. The World Health Organization announced that the dangers posed by antibiotic resistance have reached a serious threat level [3]. In addition, the US Centers for Disease Control and Prevention reported that people globally are at risk through the paradigm shift to a “post-antibiotic era” [4]. Antibiotic susceptibility testing (AST) is becoming increasingly important to counter this risk of antibiotic-resistant bacteria, not only in humans but also in animals [5]. AST is a clinical method for detecting bacterial resistance or observing the susceptibility of drugs as opposed to pathogens, enabling determination of the minimal inhibitory concentration (MIC) in each case [6]. Several AST methods have been established, such as the disk diffusion method based on solid-plate AST, the broth dilution method for liquid AST, and polymerase chain reaction-based methods [7]. Recently, numerous rapid AST methods based on microfluidic systems with microscopic analysis have been developed to reduce the time for AST and to increase the accuracy, but the inoculum effects of those methods have not been studied [8,9,10,11,12]. 

The inoculum effect is a phenomenon that is explained as an increase in MIC, which depends on the increase in the inoculated density of pathogens [13,14]. From a clinical perspective, the inoculum effect is very important because MIC values can be overestimated due to it. As a result of this, insufficient amounts of antibiotics can be administered, which would increase the mortality rate of patients [15]. In addition, owing to the overuse and misuse of antibiotics, bacterial strains are more likely to gain resistance to antibiotics, facilitating the emergence of highly resistant pathogens [16]. Given the development of new AST methods based on a microfluidic system using microscopic analysis, it is important to study the inoculum effect with these methods to prevent the associated misuse of antibiotics. 

Here, we investigate the inoculum effect by image-based AST (IBAST), which is based on a microfluidic biochip. Although conventional AST methods require 16–20 h, as well as being labor-intensive and inconvenient, IBAST can be done in only 4–6 h, depending on the bacterial strain. In addition, the system utilizes an automated microscope, which saves a lot of time and labor compared with conventional AST methods [12,17]. As a rapid AST with an automated imaging system, this method has good potential to be used clinically. When we study the inoculum effect, we also change the volume of the culture medium with the antibiotic to investigate its effect on the inoculum effect. Consequently, IBAST was conducted against two Gram-negative strains, *P. aeruginosa* ATCC 27853 and *E. coli* ATCC 25922, and two Gram-positive strains, *S. aureus* ATCC 29213 and *E. faecalis* ATCC 29212, using five different antibiotics, namely, ampicillin, cefotaxime, piperacillin, levofloxacin, and amikacin, at three different bacterial concentrations: 5 × 10^5^, 5 × 10^6^, and 5 × 10^7^ CFU/mL. We also investigated the effect of the volume of the culture medium with antibiotics on the inoculum effect. This research aids the development of rapid AST based on a microfluidic system with microscopic analysis to understand the inoculum effect and utilize the system in clinical areas appropriately. 

## 2. Results

### 2.1. Inoculum Effect of Staphylococcus aureus ATCC 29213

**Figure 1:**
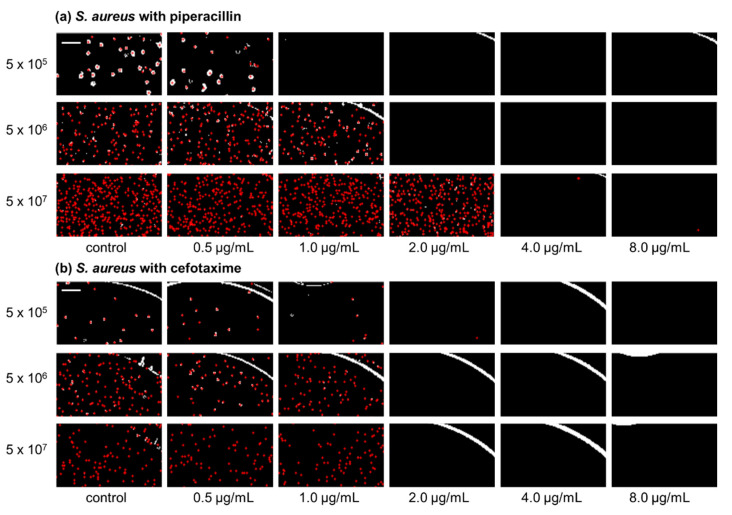
Processed images of *S. aureus* with three different concentrations, 5 × 10^5^, 5 × 10^6^, and 5 × 10^7^ CFU/mL, after 6 h of incubation exposed to piperacillin and cefotaxime. Red dots in the images illustrate the colonies of bacteria that were recognized by image processing. (**a**) Representative images of *S. aureus* with piperacillin. (**b**) Representative images of *S. aureus* with cefotaxime. The scale bar represents 200 μm.

IBAST was performed on *S. aureus* ATCC 29213 with three β-lactam antibiotics, ampicillin, cefotaxime, and piperacillin, and two non-β-lactam antibiotics, levofloxacin and amikacin. Figure 1a shows processed images of *S. aureus* ATCC 29213 of three different inocula upon 6 h of incubation after exposure to piperacillin. At 5 × 10^5^ CFU/mL, MIC was determined to be 1 μg/mL. As the bacterial density increased to 5 × 10^6^ CFU/mL and 5 × 10^7^ CFU/mL, MIC increased to 2 and 4 μg/mL, respectively. Therefore, Figure 1a demonstrates the significant inoculum effect of *S. aureus* to piperacillin. In contrast, Figure 1b shows processed images of *S. aureus* exposed to cefotaxime after incubation for 6 h. As seen in the images, MIC was identical at 2 μg/mL and there were no changes of it among the three different bacterial concentrations.

*S. aureus* ATCC 29213 was also monitored after 6 h of incubation with different antibiotics. In a normal volume of the culture medium of 90 μL, the MIC values were identical in the case of cefotaxime (Table 1). When the bacterial density was increased tenfold from the initial concentration of 5 × 10^5^ CFU/mL, the MIC values of ampicillin, levofloxacin, and piperacillin doubled. Moreover, when the bacterial concentration was increased to 5 × 10^7^ CFU/mL, the MICs of amikacin and piperacillin doubled again, while that of ampicillin increased fourfold. On the other hand, that of levofloxacin did not change at 5 × 10^7^ CFU/mL.

To investigate the variation of MICs in more detail to represent the inoculum effect, different volumes of culture media with antibiotics were used: 45 and 135 μL. Table 1 presents the MIC values in relation to the volumes of culture media with antibiotics. In the case of ampicillin, the MIC did not change with volume at a density of 5 × 10^5^ CFU/mL. However, at 5 × 10^6^ CFU/mL inoculum, the MIC increased to twofold at 45 μL compared to 90 and 135 μL. At the 5 × 10^7^ CFU/mL inoculum, the MIC increased to 4 μg/mL at 45 μL. For levofloxacin, the MIC increased from 0.12 to 0.25 μg/mL in 5 × 10^5^ and 5 × 10^6^ CFU/mL at 90 and 135 μL. However, in the other cases, the values were static at 0.25 μg/mL, with no change. In the case of amikacin, when the bacterial density increased from 5 × 10^6^ to 5 × 10^7^ CFU/mL, all the MICs of the volumes of the culture media with antibiotics doubled. At the same bacterial concentration, the volume of the culture medium with antibiotics did not affect the MICs. In the case of piperacillin, when the bacterial density was increased tenfold from the initial level, the MICs were increased by twofold in 90 μL of culture medium. When 45 μL of the culture medium with piperacillin was injected, the MIC increased fourfold between 5 × 10^6^ and 5 × 10^7^ CFU/mL. In general, the low volume of the culture medium with antibiotics contributes to the inoculum effect in *S. aureus* with ampicillin, amikacin, and piperacillin. As shown in Table 1, when *S. aureus* was exposed to ampicillin at densities of 5 × 10^6^ and 5 × 10^7^ CFU/mL, the MIC values were decreased as the amount of the culture medium increased. In addition, at 5 × 10^5^ CFU/mL for levofloxacin and at 5 × 10^5^ CFU/mL for piperacillin, the MIC values were reduced. Given that all antibiotics used in our experiments have binding process mechanisms that are irreversible [18,19], we concluded that, if the amount of antibiotics increases, this means that the antibiotics are more likely to bind to the bacteria, which in turn leads to a decrease in the MIC.

### 2.2. Inoculum Effect of Pseudomonas aeruginosa ATCC 27853

In the case of *P. aeruginosa* ATCC 27853, all antimicrobial agents showed inoculum effects under normal conditions with 90 μL of culture medium. This pathogen was exposed to each antibiotic for 6 h. As seen in Table 2, all MICs increased twofold when the bacterial density was increased from 5 × 10^5^ to 5 × 10^6^ CFU/mL. However, when the density was increased from 5 × 10^6^ to 5 × 10^7^, the MIC of amikacin increased twofold as from 5 × 10^5^ to 5 × 10^6^, but the MIC of piperacillin increased over fourfold, while in the cases of levofloxacin and cefotaxime there was no change.

When *P. aeruginosa* was exposed to four different antimicrobial agents, namely, cefotaxime, levofloxacin, amikacin, and piperacillin, at 5 × 10^5^ CFU/mL inoculum, all the MIC values were static with different amounts of the culture medium with antibiotics (Table 2). In the cases of levofloxacin, amikacin, and piperacillin, the MIC values were doubled when the bacterial inoculum was increased tenfold from the initial concentration. Cefotaxime showed different MIC values at each amount of the culture medium with antibiotics. The MIC was decreased when the amount of culture medium with antibiotics was increased from 5 × 10^6^ to 5 × 10^7^ CFU/mL. However, when 135 μL of cefotaxime was injected, the MIC was increased again at 5 × 10^7^ CFU/mL. Levofloxacin did not change the MIC, even when the bacterial density was increased from 5 × 10^6^ to 5 × 10^7^ CFU/mL. The MIC of amikacin was doubled, and piperacillin was increased more than fourfold as the inoculum size increased from 5 × 10^6^ to 5 × 10^7^ CFU/mL, regardless of the amount of antibiotics. Overall, *P. aeruginosa* showed an inoculum effect for all four antibiotics and was not affected by the amount of the culture medium with antibiotics, except in the case of cefotaxime.

As seen in Table 2, in the case of cefotaxime, the MIC value was decreased with the volume of the culture medium at the same bacterial density: 5 × 10^6^ CFU/mL. This can be interpreted as showing that an increased amount of antibiotics influences the bactericidal effect more efficiently. Owing to the binding mechanism of antibiotics, cefotaxime has an irreversible inhibition process [19]. However, at a density of 5 × 10^7^ CFU/mL, the MIC was increased again when the inoculated antibiotics increased from 90 to 135 μL. We guessed that this was because of the increased volume of the culture medium. The antibiotics were diluted with culture medium, so that if we inoculated antimicrobial agents, we also inoculated more of the medium into the well. If the medium increased, this meant that the bacteria had good conditions to grow in, even if the overall amount of antibiotics increased. Therefore, positive effects on growth occurred, resulting in MIC augmentation.

### 2.3. Inoculum Effect of Escherichia coli ATCC 25922

*E. coli* ATCC 25922 was monitored after 4 h of incubation with antimicrobial agents (Table 3). When the pathogen was exposed to ampicillin, levofloxacin, and piperacillin, the MICs were not changed under the normal conditions of 90 μL of culture medium. However, in the case of cefotaxime, the MIC was increased at 5 × 10^6^ CFU/mL. Similarly, when amikacin was applied to *E. coli*, the MIC doubled at 5 × 10^7^ CFU/mL. 

When *E. coli* was exposed to cefotaxime with different amounts of the culture medium, the MIC changed (Table 3). At a density of 5 × 10^5^ CFU/mL, the MIC decreased as the amount of antibiotics increased from 45 to 90 μL. As the bacterial density increased tenfold, the MIC increased at doses of 90 and 135 μL. In addition, when the bacterial concentration was 5 × 10^7^ CFU/mL, the MIC significantly increased at doses of 45 and 135 μL. However, at a 90 μL dose of cefotaxime, the MIC remained at 0.12 μg/mL. In addition, amikacin was also tested with different amounts of the culture medium. The only feature that changed when amikacin was used occurred at a bacterial density of 5 × 10^7^ CFU/mL. Specifically, the MIC value at 5 × 10^7^ CFU/mL was increased twofold compared with that at 5 × 10^6^ CFU/mL. Overall, *E. coli* did not show an inoculum effect among the four antibiotics, except cefotaxime. 

As shown in Table 3, at 5 × 10^5^ CFU/mL, the MIC of cefotaxime decreased as the amount of the culture medium increased. This phenomenon is also shown in Table 2, like in the case of *P. aeruginosa* against cefotaxime at 5 × 10^6^ CFU/mL. In addition, at 5 × 10^7^ CFU/mL, the MIC was increased as the amount of antibiotics increased from 90 to 135 μL. 

### 2.4. Inoculum Effect of Enterococcus faecalis ATCC 29212

As seen in Table 4, *E. faecalis* ATCC 29212 was observed after 6 h of incubation with antibiotics. Only levofloxacin showed an inoculum effect, while the results for the other agents were static, even if the number of pathogens increased. When the bacterial organisms were increased tenfold from the initial density with levofloxacin, the MIC doubled from 5 × 10^5^ to 5 × 10^6^ CFU/mL, but it did not change from 5 × 10^6^ to 5 × 10^7^ CFU/mL. In the case of levofloxacin, different amounts of the culture medium were applied to *E. faecalis*. Among the three volumes of culture media, the MICs doubled when the bacterial density was increased tenfold from the initial concentration. However, unlike the other cases, the only change occurred at 5 × 10^7^ CFU/mL at a dose of 45 μL where the MIC was doubled again.

## 3. Discussion

IBAST is a system that combines microfluidic chips, microscopic images, and image processing technology. Through this, we can get AST results quickly compared to the conventional methods. The conventional AST method based on turbidity occurred inoculum effect, so the experiment was conducted in anticipation of this phenomenon in IBAST. As a result, there were cases of inoculum effect depending on the combination of bacteria and antibiotics, and in this case, the experiment was conducted by changing the amount of badges. 

In IBAST, *S. aureus* showed an inoculum effect against ampicillin and piperacillin (Table 1). These results may be due to the mechanism of action of *β*-lactam antibiotics, such as ampicillin and piperacillin. *β*-Lactam antibiotics bind with penicillin-binding proteins (PBPs), which inhibit cell wall construction [19]. They not only inhibit bacterial cell wall synthesis but also cause morphological changes. For instance, the filamentous elongation or swelling of bacteria occurs when *β*-lactams are exposed; this results in cell lysis, eventually leading to cell death. In accordance with this, the MIC results of ampicillin and piperacillin against *S. aureus* showed clear inoculum effect according to the inoculum size (Table 1). This corresponds with the findings of several other experiments [20,21]. However, although cefotaxime is one of the *β*-lactam antibiotics, it did not show any inoculum effect in *S. aureus* in IBAST, which is in agreement with other previous experiments [22,23]. Previous studies showed that, when the bacterial inoculum increased, the MIC of cefotaxime tended not to increase [22,23]. These results may be due to the antibiotic class of cefotaxime. While ampicillin and piperacillin are within the penicillin group, cefotaxime is a subclass of cephalosporins. Given their inhibition of cell wall construction, the two antibiotics broadly have the same mechanism of action, but they have different PBP affinities. Mizuguchi et al. showed the difference of PBP affinity between ampicillin and cefazolin, which is one of the cephalosporins like cefotaxime [24]. In the case of non-*β*-lactam antibiotics on *S. aureus*, amikacin showed an inoculum effect (Table 1). Amikacin is a subclass of aminoglycosides, which are known as inhibitors of protein synthesis. The primary target of amikacin is the 30S ribosomal subunit, which is necessary for protein synthesis. Unlike tetracyclines, aminoglycoside inhibition is irreversible, which means that if binding has been completed, recycling is not possible. Therefore, the bacterial densities were found to depend on the antibiotic concentration [20,25]. Studies have suggested that this may be due to natural selection. As the bacterial density increases, it will enhance the possibility of bacterial subpopulations gaining resistance, and eventually a number of mutations will emerge, reducing antibiotic susceptibility. Levofloxacin also showed an inoculum effect against *S. aureus* (Table 1). Levofloxacin belongs to the subclass of fluoroquinolones, which are DNA synthesis inhibitors whose primary target is DNA gyrase. Levofloxacin was proven to be effective against Gram-positive and Gram-negative bacteria. Furthermore, in the present study, the MICs of levofloxacin against Gram-positive bacteria were significantly similar to those of ciprofloxacin. Several studies have shown that ciprofloxacin had a weak inoculum effect, as did levofloxacin [26,27], which is in agreement with our experimental results shown in Table 1.

*P. aeruginosa* showed an inoculum effect in all cases, regardless of the antibiotic (Table 2). In the case of piperacillin, a subclass of *β*-lactam antibiotics, previous studies also indicated that it showed an inoculum effect against *P. aeruginosa* [28,29]. When pathogens are present in a large number, they are more likely to survive because of the resistant mutants that develop via selection. Because of the combined production of *β*-lactamase, the bacteria become less susceptible to the antibiotics because the agents are broken down by inactivating the enzymes of other antibiotic-targeted pathogens. In addition, the preferential affinity of PBPs could be another possible reason for this. In this regard, the augmentation of bacterial concentration may cause inadequate saturation of the protein binding sites, which can result in a bactericidal effect. In our experiment, as shown in Table 2, at bacterial density of 5 × 10^7^ CFU/mL, we observed an abrupt increase in the MIC. Moreover, even if the bacterial growth was inhibited and there was potentially a decrease in the count of viable pathogens, they were still metabolically active. Therefore, the entire bacterial mass increased via morphological changes such as elongation. As such, the MIC result can be recognized as significantly high at a density of 5 × 10^7^ CFU/mL. In the case of cefotaxime, several studies have indicated that it showed an inoculum effect [29,30,31,32]. A large difference of MIC values was also previously identified, which agreed with our results. These results may be due to the mechanism of action of *β*-lactam antibiotics and can be explained in the same way as for piperacillin. Amikacin showed an inoculum effect against *P. aeruginosa* in IBAST. Unlike piperacillin, amikacin is a subclass of aminoglycosides, so it does not have any *β*-lactam rings in its chemical structure. Meanwhile, an inoculum effect is largely related to the inactivation of antibiotics by *β*-lactamase enzymes. Therefore, amikacin showed less inoculum effect in comparison with piperacillin, which is an agreement with our experiment [28,33]. Levofloxacin showed a limited inoculum effect against *P. aeruginosa*. With respect to non-*β*-lactam antibiotics, the pattern of the MIC values of levofloxacin is like that of amikacin. As we mentioned previously, levofloxacin is a subclass of fluoroquinolones and its activity is like that of ciprofloxacin [26,34,35,36]. Therefore, it was concluded that fluoroquinolones were little affected by inoculum size, which corresponded with our experiment. In addition, another study showed that ciprofloxacin had little inoculum effect [37]. 

*E. coli* is associated with an inoculum effect in the case of cefotaxime (Table 3). Several studies have shown that when *E. coli* was exposed to cefotaxime, the MIC tended to increase [38,39,40]. Two factors can explain the in vitro activity of the *β*-lactam drug with different inoculum sizes. The first is the intrinsic activity of the antimicrobial agents against the inoculated pathogens. The second is that susceptibility of the antibiotics to hydrolysis by *β*-lactamase of the pathogens. In other words, it was proposed that the inoculum effect depends on the amount of hydrolysis. However, another study clarified that *β*-lactamase activity was not the only factor behind the inoculum effect [38]. Moreover, it was suggested that *β*-lactam antibiotics can exhibit different degrees of morphological changes with different pathogens, which may result in different degrees of inoculum effect. In this regard, we concluded that *E. coli* has an inoculum effect against cefotaxime. Our results agree with those of other experiments showing moderate inoculum effects of cefotaxime [38,39,40]. Amikacin also showed an inoculum effect in IBAST. Amikacin, a subclass of aminoglycoside, combines with the 30S ribosomal subunit, which is an irreversible process. Owing to this characterization, we concluded that *E. coli* has a moderate inoculum effect against amikacin, which corresponds with the findings of other studies [20,25,41]. Unlike cefotaxime, ampicillin, and piperacillin did not show a significant inoculum effect in IBAST. According to previous studies, ampicillin had very little or no inoculum effect and piperacillin showed a moderate inoculum effect [15,42]. In one of these studies, it was asserted that there are two possible explanations for this: antibiotic destruction by *β*-lactamases and morphological changes such as filamentous transformation with continued growth [42]. The results of IBAST for ampicillin corresponded to the findings of other studies, while the results for piperacillin did not [20,39,42]. Neither of these antibiotics changed the MIC values. Moreover, in our experiment, we determined the MIC as 80% inhibition compared with that for a control well. We guessed that this evaluation standard may result in different results compared with other studies. In addition, penicillin and piperacillin are subclasses of penicillin, so we guessed that these two antimicrobial agents have different PBP affinities from cefotaxime, which may cause different results for the MIC. While levofloxacin had no inoculum effect against *E. coli* in IBAST, it had a minimal inoculum effect against *E. coli* in previous study [41]. We guessed that the fact that the determination of the MIC by IBAST involves counting the colonies from a single bacterium and the 80% evaluation standard would impact on the calculation of the MIC during image processing. 

*E. faecalis* is a Gram-positive bacterium resembling *S. aureus*. However, unlike *S. aureus*, *E. faecalis* had no inoculum effect against ampicillin and piperacillin, as shown in Table 4. These two are *β*-lactam antibiotics, which are subclasses of penicillin. We guessed that penicillin tends not to be affected by the inoculum size of *E. faecalis*. In the previous study, experiments on the inoculum effect of *E. faecalis* against ampicillin and piperacillin were conducted [43], the results of which do not agree with our results. We concluded that these different results could be due to the method of determining the MIC in IBAST or due to the different isolated strains of bacteria. We used the *E. faecalis* ATCC 29212 strain, but other studies did not. While one study used the *Enterococcal* strain HH22 [44], another study utilized *Enterococcus faecalis* with resistance to aminoglycoside and showing *β*-lactamase-producing ability [45], and another study also used *β*-lactamase-producing strains. On the other hand, *E. faecalis* presents a minimal inoculum effect against levofloxacin. As we discussed before, levofloxacin exhibits activity similar to that of ciprofloxacin. According to previous studies, ciprofloxacin had an inoculum effect against *E. faecalis*. Therefore, we concluded that levofloxacin may have an inoculum effect against *E. faecalis*, which is in agreement with previous study [37,43].

## 4. Materials and Methods

### 4.1. Fabrication of the IBAST Chip

**Figure 2:**
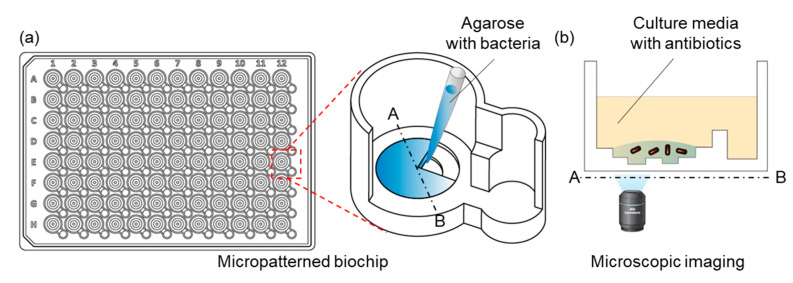
(**a**) Schematics of micropatterned biochip based on 96-well plate format. Detailed structure of a single well, containing a main well and a side well. The larger main well is inoculated with a mixture of bacteria and agarose. The smaller side well is for antimicrobial agents with MHB medium. (**b**) Schematic of cross-section view of testing well. Antibiotic solutions diluted with MHB are applied to the side well, after which diffusion occurs toward the main well. Bacteria are fixed with agarose and the culture media with antibiotic diffuse into the agarose.

The IBAST chip was based on a 96-well plate format and was designed using 3D CAD software (SolidWorks v2014; Dassault Systemes SolidWorks Corp., Vélizy-Villacoublay, France). This micropatterned chip was made by injection molding process (NT2–120; Woojin Plaimm, Republic of Korea) of polystyrene (K-RESIN; Chevron Phillips Chemical, Baytown, TX, United States) (Figure 2a). In advance of IBAST, air plasma treatment (CUTE-MP; FemtoScience, Hwaseong-si, Republic of Korea) was performed for 1 min to provide hydrophilicity to the chips, which helps uniform distribution of agarose mixture on the chip.

### 4.2. Stock Preparation and Subculture of Bacteria

Four standard bacterial strains (*P. aeruginosa* ATCC 27853, *E. coli* ATCC 25922, *S. aureus* ATCC 29213, and *E. faecalis* ATCC 29212) of Clinical and Laboratory Standards Institute (CLSI) were purchased from MicroBioLogics, Inc. (Saint Cloud, MN, USA). We conducted all the experiments in accordance with the ethical standards from American Type Culture Collection and performed all the experiments in the biosafety cabinet. Bacterial stock solutions were made by mixing 50 mL of glycerol (Sigma-Aldrich, St. Louis, MO, USA) and 50 mL of deionized water. A total of 400 μL of 50% glycerol-deionized water mixture was mixed with 800 μL of bacterial cation adjusted Mueller–Hinton broth (CAMHB; BD Biosciences, San Jose, CA, USA) mixture. These stock solutions were placed in microcentrifuge tubes (SPL Life Sciences, Seoul, Korea) and cryopreserved at −70 °C in a deep freezer. For the IBAST, subcultures were performed before the experiment. Stock solutions from aliquots were inoculated with one loop on Luria–Bertani agar plates (KisanBio Co., Ltd., Seoul, Korea) and were incubated in a 37 °C incubator overnight. After 20 to 24 h of incubation, several colonies were formed and used to generate target concentration of bacteria. 

### 4.3. Antibiotic Preparation 

Five antimicrobial agents were used to determine the MIC values of each bacterium. Three *β*-lactam antibiotics were used: ampicillin, cefotaxime, and piperacillin (Sigma-Aldrich). In addition, levofloxacin (Sigma-Aldrich), a representative quinolone, and amikacin (Sigma-Aldrich), a representative aminoglycoside, were used. These antibiotics were diluted with deionized water to make aliquots and stored at −70 °C in a deep freezer. Each aliquot was made up to a concentration of 10 mg/mL. For the IBAST and to accurately determine the MIC values, these stock solutions were adjusted by mixing MHB medium to match the concentrations of quality control range of CLSI, so that twofold dilutions were made.

### 4.4. Inoculum Sizes of Bacteria 

After incubation, four CLSI standard strains of bacteria were diluted to three concentrations each. To measure inoculum effects, bacterial colonies on agar plates were collected in a loop and mixed with MHB medium to make McFarland 0.5 at approximately 1.5 × 10^8^ CFU/mL. The cell density was calibrated using ultraviolet spectrophotometric devices (DensiCHEK™; bioMerieux Inc., Marcy l’Étoile, France). Then, the suspension was mixed with different amounts of MHB medium to achieve concentrations of 1.5 × 10^7^, 1.5 × 10^6^, and 1.5 × 10^5^ CFU/mL. A total of 200 μL of bacterial solution was mixed with 600 μL of 0.5% agarose in a liquid state at 37 °C. Then, 10 μL of bacterial mixtures were inoculated in each main well of micropatterned chip, followed by solidification at room temperature. A total of 90 μL of antimicrobial agents in culture medium were injected into each side well (Figure 2a). Antibiotics and culture medium diffused toward the bacteria, which was captured using a charge coupled device camera with a 20× microscopic lens (Figure 2b). After the inoculation, the micropatterned chip was incubated in the chamber at 37 °C. In the case of *E. coli* ATCC 25922, incubation was performed for 4 h. Other pathogens such as *P. aeruginosa* ATCC 27853, *S. aureus* ATCC 29213, and *E. faecalis* ATCC 29212 were incubated for 6 h. Imaging was performed immediately after the incubation, utilizing an automated microscope. Subsequently, all MIC values were compared with results obtained by the broth microdilution (BMD) method to determine whether the results were reliable.

### 4.5. Determination of Growth of Bacteria

In the IBAST experiment, growth was determined by comparing its bacterial area with a threshold of 20% of the growth in control wells containing only culture medium without antibiotics. This standard was also applied to the broth microdilution test.

### 4.6. Image Processing

**Figure 3:**
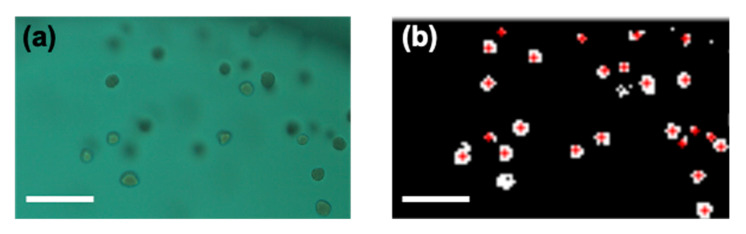
(**a**) RGB raw image from charge coupled device (CCD)camera. (**b**) Processed binary image with red crosses indicating the bacterial colonies.

A 20× microscope objective lens was used for imaging and the field of view of one image was 1126.4 × 594 μm. Microfabricated chips were inserted into a microscope device (QuantaMatrix, Inc., Seoul, Korea) and monitored using a complementary metal–oxide–semiconductor camera. To accurately determine the MIC values of each case, images were processed digitally. With the help of MATLAB (version 2019; Natick, MA, USA), the image processing program was coded. RGB raw images were then changed to binary format in order to count the area of bacterial colonies in the image (Figure 3). Then, black and white inversion was performed, so that the background of each image could be discriminated from pathogens. Specifically, the pixel number of white area was counted as the area of bacterial colony. If the imaging data of bacterial growth were lower than 20% compared with the control well, the result was recorded as no growth. Red dots in Figure 3b represent colonies recognized by image processing. In addition, to remove a main well line that presented a shape like a curve at the far right end, a parameter of circularity was used. If the circularity was lower than 0.3, MATLAB eliminated the curve line and did not count it as a bacterium.

## 5. Conclusions

In this study, we demonstrated IBAST, which is a method of rapid AST utilizing microfluidic chip and microscopic imaging. The entire AST process takes 4–6 h, enabling the fast determination of the proper antibiotics for the patients. It also helps to accurately determine the MIC, considering the inoculum effect. While conventional methods depend on optical density by checking turbidity, IBAST can monitor the colony formation of single bacteria in more detail. Overall, IBAST showed similar patterns of inoculum effect compared with conventional methods. However, in some cases, IBAST showed a different pattern of inoculum effect, due to the different method of determination of the growth of bacteria. To apply IBAST in a clinical context, broad studies related to the inoculum effect are needed. After that, the IBAST system can be applied in numerous clinical environments to avoid the overuse or misuse of antibiotics and save numerous patients from severe diseases.

## Figures and Tables

**Table 1:** Minimal inhibitory concentration (MIC) values of *S. aureus* according to different bacterial inoculum sizes and volumes of culture media with antibiotics. The unit of MIC is μg/mL.

Antibiotics		Ampicillin			Cefotaxime			Levofloxacin			Amikacin			Piperacillin		
Inoculum size (CFU/mL)		5 × 10^5^	5 × 10^6^	5 × 10^7^	5 × 10^5^	5 × 10^6^	5 × 10^7^	5 × 10^5^	5 × 10^6^	5 × 10^7^	5 × 10^5^	5 × 10^6^	5 × 10^7^	5 × 10^5^	5 × 10^6^	5 × 10^7^
Volume of culture media w/antibiotics (μL)	45	0.25	1	4	−	−	−	0.25	0.25	0.25	1	1	2	1	2	8
	90	0.25	0.5	2	2	2	2	0.12	0.25	0.25	1	1	2	1	2	4
	135	0.25	0.5	2	−	−	−	0.12	0.25	0.25	1	1	2	1	2	2

**Table 2:** MIC values of *P. aeruginosa* according to different bacterial inoculum sizes and volumes of culture media with antibiotics. The unit of MIC is μg/mL.

Antibiotics		Cefotaxime			Levofloxacin			Amikacin			Piperacillin		
Inoculum size (CFU/mL)		5 × 10^5^	5 × 10^6^	5 × 10^7^	5 × 10^5^	5 × 10^6^	5 × 10^7^	5 × 10^5^	5 × 10^6^	5 × 10^7^	5 × 10^5^	5 × 10^6^	5 × 10^7^
Volume of culture media w/antibiotics (μL)	45	8	32	32	0.5	1	1	0.5	1	2	2	4	>16
	90	8	16	16	0.5	1	1	0.5	1	2	2	4	>16
	135	8	16	>64	0.5	1	1	0.5	1	2	2	4	>16

**Table 3:** MIC values of *E. coli* according to different bacterial inoculum sizes and volumes of culture media with antibiotics. The unit of MIC is μg/mL.

Antibiotics		Ampicillin			Cefotaxime			Levofloxacin			Amikacin			Piperacillin		
Inoculum size (CFU/mL)		5 × 10^5^	5 × 10^6^	5 × 10^7^	5 × 10^5^	5 × 10^6^	5 × 10^7^	5 × 10^5^	5 × 10^6^	5 × 10^7^	5 × 10^5^	5 × 10^6^	5 × 10^7^	5 × 10^5^	5 × 10^6^	5 × 10^7^
Volume of culture media w/antibiotics (μL)	45	−	−	−	0.12	0.12	>0.25	−	−	−	0.5	0.5	1	−	−	−
	90	8	8	8	0.06	0.12	0.12	0.015	0.015	0.015	0.5	0.5	1	4	4	4
	135	−	−	−	0.06	0.12	>0.25	−	−	−	0.5	0.5	1	−	−	−

**Table 4:** MIC values of *E. feacalis* according to different bacterial inoculum sizes and volumes of culture media with antibiotics. The unit of MIC is μg/mL.

Antibiotics		Ampicillin			Levofloxacin			Piperacillin		
Inoculum size (CFU/mL)		5 × 10^5^	5 × 10^6^	5 × 10^7^	5 × 10^5^	5 × 10^6^	5 × 10^7^	5 × 10^5^	5 × 10^6^	5 × 10^7^
Volume of culture media w/antibiotics (μL)	45	−	−	−	0.5	1	2	−	−	−
	90	1	1	1	0.5	1	1	2	2	2
	135	−	−	−	0.5	1	1	−	−	−

## Data Availability

The data presented in this study are available on request from the corresponding author.
